# Supplementary material for: Heparin-based hydrogel scaffolding alters the transcriptomic profile and increases the chemoresistance of MDA-MB-231 triple-negative breast cancer cells
Source: Biomater Sci. 2020 Feb 13;8(10):2786–96. doi: 10.1039/c9bm01481k (PMC7497406; doi:10.1039/c9bm01481k)
Supplement: Supplementary file 2 [file BM-008-C9BM01481K-s002.zip › Supplementary File 4/EGFvControl/Pathways/my_analysis.Gsea.1545200981068/heat_map_corr_plot.html]

Heat map and correlation plot for expr.class.cls#EGF\_versus\_CONTROL  

Fig 1: heat\_map      
 Heat Map of the top 50 features for each phenotype in expr.class.cls#EGF\_versus\_CONTROL

  
  

Fig 2: Ranked Gene List Correlation Profile      
 Ranked list correlations for expr.class.cls#EGF\_versus\_CONTROL

  
  
    
